# The Putative Smallest Introns in the *Arabidopsis* Genome

**DOI:** 10.1093/gbe/evy197

**Published:** 2018-09-01

**Authors:** Wenzhen Cheng, Yunlin Zhou, Xin Miao, Chuanjing An, Hongbo Gao

**Affiliations:** College of Biological Sciences and Technology, Beijing Forestry University, China

**Keywords:** *Arabidopsis*, very small introns, reverse transcription PCR

## Abstract

Most eukaryotic genes contain introns, which are noncoding sequences that are removed during premRNA processing. Introns are usually preserved across evolutionary time. However, the sizes of introns vary greatly. In *Arabidopsis*, some introns are longer than 10 kilo base pairs (bp) and others are predicted to be shorter than 10 bp. To identify the shortest intron in the genome, we analyzed the predicted introns in annotated version 10 of the *Arabidopsis thaliana* genome and found 103 predicted introns that are 30 bp or shorter, which make up only 0.08% of all introns in the genome. However, our own bioinformatics and experimental analyses found no evidence for the existence of these predicted introns. The predicted introns of 30–39 bp, 40–49 bp, and 50–59 bp in length are also rare and constitute only 0.07%, 0.2%, and 0.28% of all introns in the genome, respectively. An analysis of 30 predicted introns 31–59 bp long verified two in this range, both of which were 59 bp long. Thus, this study suggests that there is a limit to how small introns in *A. thaliana* can be, which is useful for the understanding of the evolution and processing of small introns in plants in general.

## Introduction

Introns are important features of eukaryotic genes. They are usually noncoding sequences in the gene and have to be removed from the premRNA (Roy and Gilbert 2006). The boundary sequences of introns are usually conserved with GU in the 5′ end and AG in the 3′ end, suggesting that they may be important for intron splicing in premRNA (Mishra and Thakran 2018). Introns are classified into several types and also exist in the genes of chloroplasts, mitochondria, and bacteria (Harris and Breaker 2018; He et al. 2018; Qu et al. 2018). The most common type of introns is the type I intron, which exists in most nuclear genes in eukaryotes. The splicing process involves two steps of transesterification, and the branch point is A. In the first step, the 5′ end of intron is cut and connected to the branch point. In the second step, the 3′ end of intron is cut, the exons are joined and the intron is released as lariat (Wilkinson et al. 2018).

Introns are preserved during evolution, which makes them important in the study of genomics (Russell et al. 2005; Roy and Gilbert 2006; Bulman et al. 2007). They have multiple functions in the cells, including the regulation of gene expression and the increase of protein diversity by alternative splicing (Wieringa et al. 1984; Kriventseva et al. 2003; Stetefeld and Ruegg 2005; Mishra and Thakran 2018; Shepelev et al. 2018). Entire intron sequences are not conserved, making it easy for them to accumulate mutations (Frigola et al. 2017). The sizes of introns range from very large—dozens of kilo base pairs (kbp)—to minute—tens of bp. During evolution, introns show signs of extension and retraction. We have shown that transposable elements and large indels could be the causes of this phenomenon for large introns in *Arabidopsis* (Chang et al. 2017). However, most of the introns are not large, that is, introns a few hundred bp long and even shorter are more common in *Arabidopsis* (Arabidopsis Genome Initiative 2000).

The smallest exon in *Arabidopsis* was found to be 1 bp (Guo and Liu 2016). To determine the smallest introns in *Arabidopsis* and understand the mechanism of intron retraction of small introns, we analyzed the very small predicted introns in *Arabidopsis*. There are 103 introns of 30 bp or shorter in the TAIR 10 annotation of the *Arabidopsis* genome, and this constitutes only 0.08% of all the introns in the genome. However, a detailed bioinformatics and experimental analysis found no evidence for the existence of these very small predicted introns in *Arabidopsis*. A further analysis of 30 selected introns between 30 bp and 60 bp verified two introns with a size of 59 bp. These results give some useful implications for our understanding of plant genomes.

## Materials and Methods

### Materials

The plant materials used in this study were Columbia-0 (Col-0) ecotype *Arabidopsis thaliana* plants grown at 21 °C and under 16 h light and 8 h dark cycle conditions. Whole plants of two-week-old seedlings, leaves of four-week-old plants and floral tissues were used to isolate RNA. Total RNA was isolated with a RNeasy Plant Kit (Aidlab, Beijing, China).

### Bioinformatics Analysis

Information about the introns in *Arabidopsis* was obtained from the TAIR website (https://www.arabidopsis.org; last accessed September 11, 2018). The detailed structure information on individual genes was also searched and checked manually on the TAIR website. Gene expression levels at different developmental stages were checked by looking up the microarray data (http://jsp.weigelworld.org/expviz/expviz.jsp; last accessed September 11, 2018; supplementary table S1, Supplementary Material online). Statistical analysis of the length of introns and the expression levels of the corresponding genes was carried with Microsoft Excel.

### RT-PCR and Sequence Analysis

For RT-PCR analysis, mRNA was first reverse transcribed with the RevertAid First Strand cDNA Synthesis Kit (Fermentas, Waltham, MA, USA). cDNA was amplified with the corresponding primers (supplementary table S2, Supplementary Material online) by Taq DNA polymerase. The PCR products were run on agarose gel and recovered for DNA sequencing. The sequencing results were analyzed by BLAST search on the TAIR website (https://www.arabidopsis.org/Blast/index.jsp; last accessed September 11, 2018) and calculated manually one by one.

## Results

### Selection of Very Small Introns for Analysis

According to data shown in the TAIR 10.0 annotation of the *Arabidopsis* genome and our previous analysis (Chang et al. 2017), the *Arabidopsis* genome has 62,565 introns that are shorter than 100 bp, and these constitute 48.93% of all the introns in the genome. Of these, 20,395 are 90–99 bp, 26,585 are 81–89 bp, and 13,050 are 71–80 bp. Overall, introns from 70 bp to 99 bp constitute 95.9% of the introns shorter than 100 bp. The number of introns from 60 bp to 69 bp is 1,738, which is much less. Furthermore, there were 357, 253, and 93 introns found to be 50–59 bp, 40–49 bp, and 30–39 bp in length, respectively.

There are 9 introns 30 bp long and 94 introns shorter than 30 bp. These account for only 0.16% of the introns shorter than 100 bp. We chose these very small predicted introns for further analysis and verification by RT-PCR and sequence analysis to determine whether they are indeed true introns or are actually annotation artifacts.

**Fig. 1.**
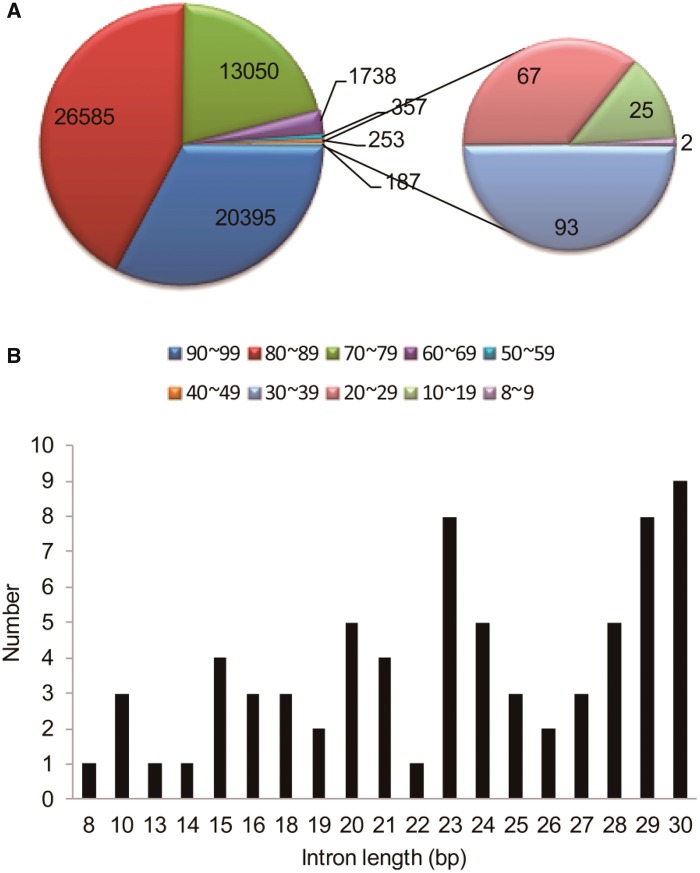
—Distribution of the predicted introns shorter than 100 bp. (*A*) In the *Arabidopsis* genome, there are 62,565 introns shorter than 100 bp. A classification of these introns based on the size is shown. Numbers of introns 50–59 bp and 40–49 bp in length are 357 and 253, respectively. (*B*) The length of introns 30 bp or shorter and the number of introns of that length, as predicted by TAIR.

**Fig. 2.**
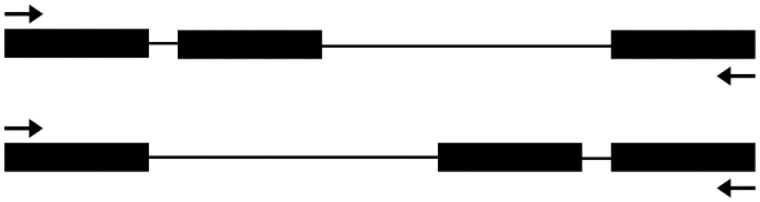
—A diagram of the principle for the RT-PCR analysis primers. Besides the putative very small intron, another intron was also included in the RT-PCR analysis to make sure that the PCR product is from a true cDNA fragment. Black boxes represent the exon, and black lines represent the intron. Upper: the very small intron is before another larger intron; lower: the very small intron is after another larger intron. The arrows indicate the positions of the primers for RT-PCR analysis.

**Fig. 3.**
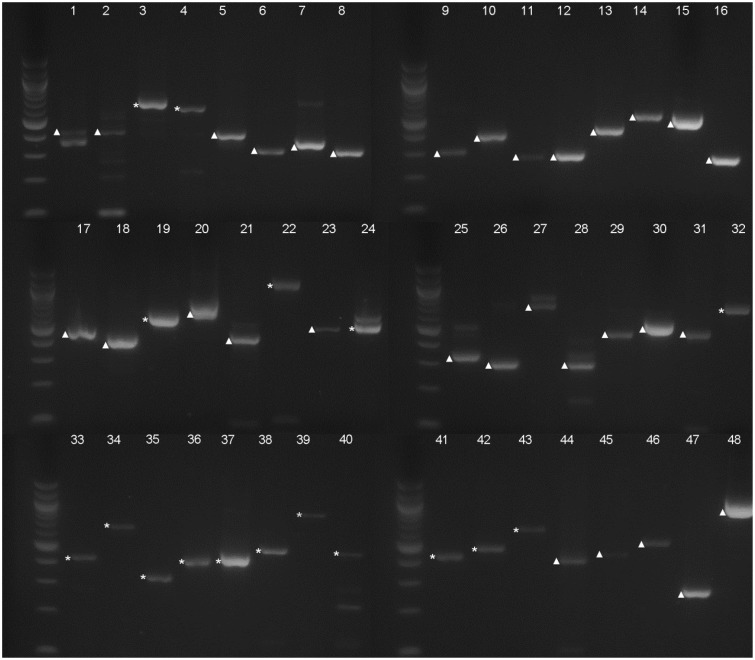
—Electrophoresis analysis of the RT-PCR products of the selected 48 predicted introns. Each number (1–48) corresponds with one predicted intron from RT-PCR analysis, also shown in table 1. Bands of cDNA are marked with ▲; bands of genomic DNA are marked with *. Molecular weight markers are 100 bp DNA ladders (New England Biolabs).

According to the new version of the annotated *Arabidopsis* genome (Araport11, which is partially released), some of these 103 very small introns were removed, leaving only 71. The distribution of their lengths is shown in figure 1*B*. RT-PCR and sequencing were used to verify whether these are true introns. At first, primers were designed for RT-PCR in a way that they should be able to amplify a true cDNA fragment to show the existence of not only the very small predicted introns for verification but also another intron upstream or downstream (fig. 2). This way, we could judge whether the PCR product comes from the real transcript or genomic DNA contamination. This is because a spliced piece of intron sequence will determine if a sequence is cDNA. Otherwise, the sequence may come from genomic DNA. Sixteen introns were excluded from our analysis based on this criterion because they are the only predicted intron in the gene. Six of those introns were checked and their sequences were included in the PCR products, and we excluded them because we were not sure whether these PCR products were amplified from genomic DNA or cDNA (data not shown). Seven other introns were also excluded because their neighboring exons were not suitable for designing primers. The remaining 48 introns were numbered 1, 2, 3, and etc. in the analysis (fig. 3 and table 1).

**Table 1:** Some Basic Information of the Very Small Introns Analyzed in This Study

No.	Predicted Intron	Size (bp)	Sequencing Results	Existence	No. of Splice Variants	Homologous Genes
1	AT1G62580.1-5	27	cDNA	No	3	*AT1G63340*, *AT1G12200*
2	AT2G04395.1-2	29	cDNA	No	5	*AT2G05210*
3	AT5G51795.1-2	28	gDNA	UJ	1	*AT1G55460*, *AT3G29075*
4	AT2G07240.1-4	30	gDNA	UJ	1	No homolog
5	AT2G21330.3-6	30	cDNA	No	3	*AT4G38970*, *AT2G01140*
6	AT2G44980.1-10	30	cDNA	No	3	No homolog
7	AT5G50080.1-1	27	cDNA	No	2	*AT2G47520*, *AT5G64750*, *AT5G47220*
8	AT3G53740.1-3	27	cDNA	No	4	*AT2G37600*, *AT5G02450*
9	AT2G41700.2-18	30	cDNA	No	2	No homolog
10	AT2G31370.5-6	28	cDNA	No	7	*AT1G06070*
11	AT1G51490.1-10	23	cDNA	No	1	No homolog
12	AT3G51260.2-3	21	cDNA	No	2	*AT5G66140*
13	AT3G55280.3-3	18	cDNA	No	3	*AT2G39460*
14	AT4G35300.3-3	30	cDNA	No	11	No homolog
15	AT1G01620.2-1	29	cDNA	No	2	*AT4G00430*, etc.^a^
16	AT3G53980.2-2	25	cDNA	No	2	*AT5G05960*
17	AT3G59350.3-6	23	cDNA	No	6	*AT2G43230*, etc.^b^
18	AT2G05520.2-2	21	cDNA	No	6	*AT2G05380*, etc.^c^
19	AT2G10930.1-1	29	gDNA	UJ	1	*AT5G48500*
20	AT4G38300.1-2	28	cDNA	No	1	*AT4G38650*
21	AT1G71280.1-2	25	cDNA	No	2	*AT1G71370*, *AT5G05450*
22	AT3G28170.1-1	10	gDNA	UJ	1	No homolog
23	AT1G18050.1-3	8	cDNA	No	1	No homolog
24	AT5G22050.1-7	20	gDNA	UJ	2	No homolog
25	AT2G40920.2-1	16	cDNA	No	2	*AT2G40910*, *AT2G40780*
26	AT1G27290.2-2	16	cDNA	No	2	No homolog
27	AT1G02950.3-4	15	cDNA	No	5	No homolog
28	AT1G31170.3-5	15	cDNA	No	5	No homolog
29	AT5G48760.2-1	13	cDNA	No	2	*AT3G07110*, *AT3G24830*, *AT4G13170*
30	AT2G14720.2-1	10	cDNA	No	2	*AT2G14740*
31	AT5G30341.1-1	30	cDNA	No	1	*AT1G41890*
32	AT4G06479.1-1	29	gDNA	UJ	1	No homolog
33	AT2G13125.1-1	29	gDNA	UJ	1	*AT1G47690*, *AT1G47700*, *AT1G47680*
34	AT2G06500.1-1	29	gDNA	UJ	1	No homolog
35	AT1G49015.1-2	29	gDNA	UJ	1	*AT5G27640*, *AT5G25780*
36	AT3G28020.1-4	28	gDNA	UJ	1	*AT3G48770*
37	AT1G76720.1-13	26	gDNA	UJ	2	*AT1G76820*, *AT1G76810*, *AT2G27700*, *AT1G21160*
38	AT2G18530.1-2	24	gDNA	UJ	1	*AT3G46160*, *AT3G46180*
39	AT2G24340.1-3	24	gDNA	UJ	1	No homolog
40	AT3G27600.1-1	23	gDNA	UJ	1	No homolog
41	AT2G13125.1-2	23	gDNA	UJ	1	No homolog
42	AT2G11010.1-4	23	gDNA	UJ	1	*AT4G06608*, *AT5G29030*, *AT5G29040*
43	AT1G35860.1-1	23	gDNA	UJ	1	No homolog
44	AT2G05440.4-2	21	cDNA	No	9	*AT2G05510*, *AT2G05441*, *AT2G05380*
45	AT3G05450.1-1	19	cDNA	No	1	No homolog
46	AT1G72270.1-13	18	cDNA	No	1	*AT4G27010*
47	AT4G13850.2-5	15	cDNA	No	4	*AT5G61030*
48	AT1G24460.1-4	14	cDNA	No	2	No homolog
S1	AT1G76530.1-5	31	cDNA	80 bp	3	*AT1G76520*, *AT1G20925*
S2	AT4G01780.1-2	32	gDNA	UJ	1	No homolog
S3	AT4G28670.1-3	34	cDNA	74 bp	1	No homolog
S4	AT4G20900.1-4	35	cDNA	83 bp	2	*AT5G44330*
S5	AT5G07510.2-2	36	cDNA	No	3	*AT5G07520*, *AT5G07600*, *AT5G07540*
S6	AT2G36010.2-1	36	cDNA	516 bp	3	No homolog
S7	AT3G56300.1-5	37	cDNA	No	3	*AT5G38830*
S8	AT1G02670.1-5	37	cDNA	No	7	*AT1G05120*
S9	AT1G16150.1-2	38	cDNA	92 bp	1	*AT1G16130*
S10	AT1G14390.1-4	39	cDNA	96 bp	1	*AT2G02780*, *AT2G02765*, *AT1G14400*, *AT2G02760*
S11	AT3G13920.2-5	41	cDNA	No	5	*AT1G54270*, *AT1G72730*
S12	AT4G04710.1-3	43	cDNA	82 bp	4	*AT4G04720*, etc.^d^
S13	AT5G40600.1-1	44	cDNA	534 bp	4	No homolog
S14	AT1G19090.1-3	44	cDNA	No	1	*AT5G40380*
S15	AT4G04680.1-4	45	cDNA	No	2	*AT5G06350*
S16	AT1G48740.1-4	45	cDNA	81 bp	4	*AT1G48700*, *AT5G43660*, *AT1G48698*
S17	AT3G11040.1-9	45	cDNA	111 bp	2	*AT3G61010*, *AT5G05460*
S18	AT2G35075.1-2	46	gDNA	UJ	1	No homolog
S19	AT4G15300.1-3	46	cDNA	88 bp	3	*AT1G65670*, *AT3G30290*, *AT4G15393*
S20	AT4G14310.2-2	47	cDNA	363 bp	2	No homolog
S21	AT3G43290.1-1	51	gDNA	UJ	1	*AT4G19240*, *AT3G27906*
S22	AT3G56160.1-1	52	cDNA	133 bp	5	No homolog
S23	AT3G09090.2-12	55	cDNA	71 bp	3	No homolog
S24	AT1G15120.2-5	55	cDNA	92 bp	2	*AT2G01090*
S25	AT4G12750.1-7	56	cDNA	98 bp	1	No homolog
S26	AT4G21820.1-9	56	cDNA	102 bp	3	No homolog
S27	AT4G24930.1-3	59	cDNA	**Yes**	1	No homolog
S28	AT3G23080.2-4	59	cDNA	**Yes**	3	*AT4G14500*, *AT4G14510*
S29	AT2G30650.1-3	59	gDNA	UJ	2	No homolog
S30	AT2G29390.1-5	59	cDNA	95 bp	6	*AT1G07420*

Note.—Genes with an *E* value of 1*E*–7 or smaller in the BLAST search were seen as homlogous genes. Some of the introns were larger than predicted, so their actual sizes were shown in the table.

g DNA: genomic DNA; UJ: unable to judge, because the sequence of the PCR product is the same as the genomic DNA.

a *AT4G00430, AT3G61430, AT4G23400, AT2G45960, AT2G16850, AT4G35100, AT3G53420, AT2G37170, AT2G37180, AT4G00413, AT3G54820*.

b *AT2G43230, AT3G17410, AT2G47060, AT3G62220, AT2G30740, AT1G48210, AT1G06700, AT2G30730*.

c *AT2G05380, AT2G05530, AT2G05440, AT2G05441, AT2G05510*.

d *AT4G04720, AT4G04695, AT4G04740, AT4G04700, AT4G21940*.

### RT-PCR Analysis of Predicted Introns 30 bp or Smaller

RT-PCR of the cDNA was carried out to verify the existence of the 48 very small predicted introns. The PCR products were first analyzed by electrophoresis (fig. 3). The expression levels of these genes in different developmental stages were referred from previous microarray analyses (http://jsp.weigelworld.org/expviz/expviz.jsp; last accessed September 11, 2018). In the beginning, we amplified the genes with relatively high expression levels, then analyzed the genes with a lower expression level and those with expression close to the basal level. A few of the genes had multiple bands (fig. 3). All the major amplification products resembling the size of the cDNAs were excised from the gel and sequenced.

Our sequencing results indicated that most of the genes with detectable expression levels from the microarray data had PCR products from cDNA, whereas a large part of the genes with a very low expression level had PCR products from genomic DNA (fig. 3, supplementary table S1, Supplementary Material online). These judgements were based on the principle shown in figure 2, that is, only sequences that showed evidence of being spliced out of an intron were considered as true cDNA. Overall, cDNA from 31 genes was amplified and sequenced. However, a BLAST search against *Arabidopsis* genomic DNA showed that the very small predicted introns are all included in the cDNA sequence, suggesting that none of them exist.

Sequence analysis results indicated that intron AT2G31370.5-6 and the flanking sequences have many “CAACAG” repeats, which may cause a misprediction. During the BLAST analysis of the sequencing results, we also found that these genes with very small predicted introns often have multiple splice isoforms and homologous genes (table 1), which also probably caused misprediction.

### RT-PCR Analysis of Predicted Introns 31–59 bp Long

**Fig. 4.**
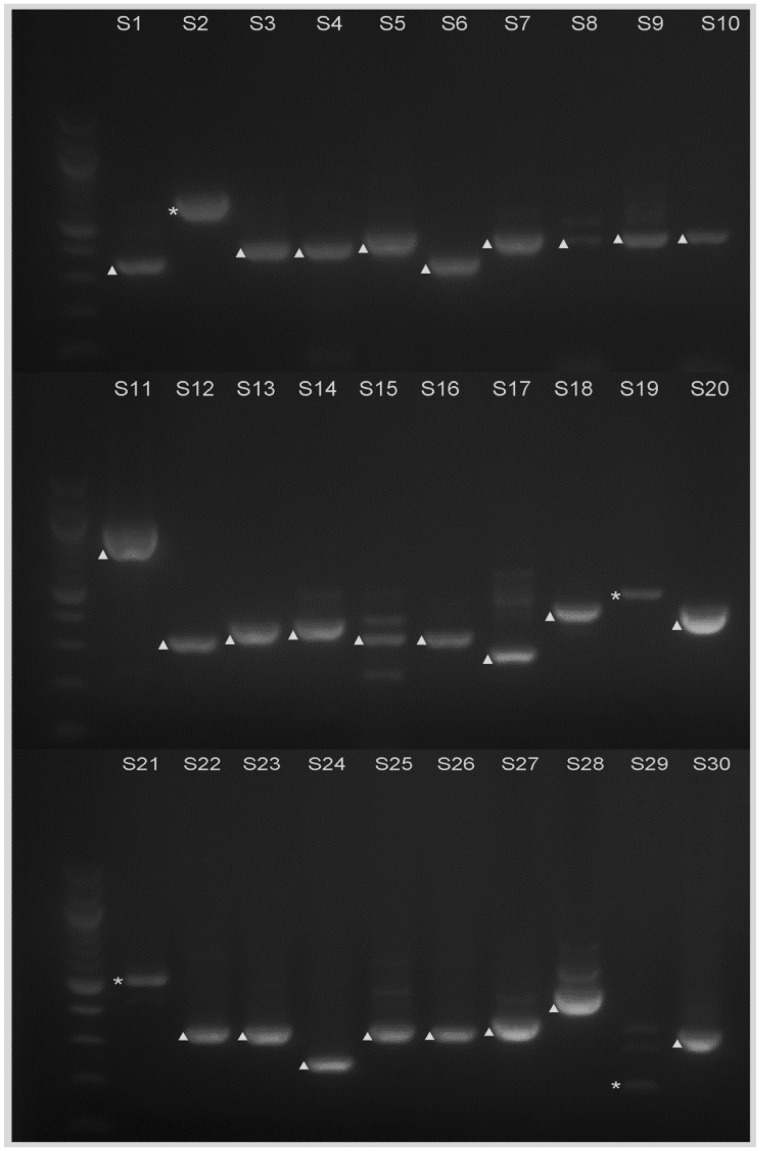
—Electrophoresis analysis of the RT-PCR products from the selected 30 predicted introns 31–59 bp in length. S1–S30 correspond to each intron that was analyzed, which are also shown in table 1. Bands of cDNA are marked with ▲; bands of genomic DNA are marked with *. Molecular weight markers are 100 bp DNA ladders (New England Biolabs).

There are 694 introns predicted to be 30–60 bp long. It is not realistic to test all the introns in this range, so we selected some of the genes with relatively high expression levels for the further analysis and exploration (table 1 and fig. 4). Thirty introns were selected in total, and labeled S1, S2, …, S30 in the analysis. Among those, six predicted introns were part of the cDNA sequences, so we concluded that they are not actually introns. Eighteen introns were predicted inaccurately; that is, a fragment either in front of or behind the intron—or sequences both in front of and behind the intron—were spliced out, so that the actual size of the intron is larger than was predicted (table 1). An additional exon and intron were also spliced out for S6 (AT2G36010.2-1), S13 (AT5G40600.1-1), and S20 (AT4G14310.2-2).

Finally, S27 (AT4G24930.1-3) and S28 (AT3G23080.2-4) were proven to be true intron. At 59 bp each, they are the smallest introns found in this study.

### Introns between 60 and 90 bp

**Fig. 5.**
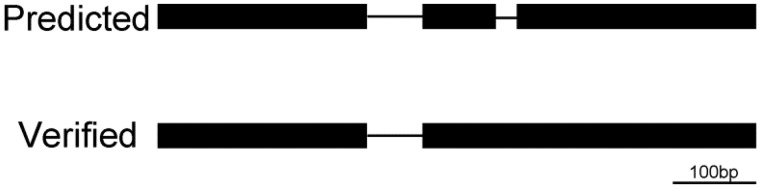
—Analysis of the introns in AT1G71280. Upper: the predicted gene model; the very small predicted intron on the right, AT1G71280.1-2, does not exist. Lower: the confirmed gene model; the intron AT1G71280.1-1 is 66 bp.

Although almost all of the very small introns were not confirmed, we found some introns that are also relatively very small. For example, in the analysis of the predicted intron AT1G71280.1-2, we used cDNA sequencing to confirm that the control intron AT1G71280.1-1 is 66 bp (fig. 5) and the control intron of AT3G55280.3-3 is 71 bp long. More introns were found to be exactly 80 bp or close to it. For example, the control introns of AT1G51490.1-10, AT2G44980.1-10, AT2G21330.3-6, AT1G02950.3-4, and AT1G31170.3-5 were 80 bp, 81 bp, 82 bp, 84 bp, and 87 bp, respectively.

## Discussion

We analyzed the small introns in the genome of *Arabidopsis* in order to find its smallest introns, and found 103 predicted introns that were 30 bp or smaller in the TAIR10 annotation and 71 in the Araport11 annotation. We narrowed these predicted introns into 48 likely candidates and a further RT-PCR analysis amplified cDNA fragments from 31 genes and genomic DNA fragments from 17 genes. However, we found no evidence that any of these candidates actually were introns. A further analysis of 30 selected introns between 30 bp and 60 bp finally verified two small introns of 59 bp.

Although the genome of *Arabidopsis* was sequenced and annotated at very high quality, it is not error free. *Arabidopsis* is a higher plant and its genome is ∼130 Mb in size, making it one of the smallest reported plant genomes (Arabidopsis Genome Initiative 2000; Bennett et al. 2003). More than 30,000 genes were predicted in *Arabidopsis*, suggesting the genome is still complicated (Arabidopsis Genome Initiative 2000; Press et al. 2018). Many of the genes we analyzed have homologs in the genome; some of them even have >10. This may interfere with the accuracy of gene prediction. That these very small introns were incorrectly predicted also indicates the misprediction of the corresponding genes. Furthermore, the presence of these sequences in the coding sequence will cause at least an insertion of the protein sequences and very likely a change in the reading frame of the genes, which will often result in premature stop codons. Seventeen out of the 48 genes with introns of 30 bp or smaller analyzed in this study were found to be PCR products of genomic DNA instead of cDNA, and these genes all have low expression levels. Therefore, these results indicate that most of these 48 genes are probably pseudogenes.

Our data suggest introns of 30 bp or smaller seem to not exist in the *Arabidopsis* genome, and this provides some useful insights into the smallest introns in plants as a whole. Introns may retract by DNA deletion, but they are usually preserved during evolution. There is probably a lower limit of the size of introns, but 30 bp seems to be too small. There are several hundreds of predicted introns with sizes from 30 to 60 bp, and these also constitute a very small percentage of the total introns in the genome. The rarity of these predicted introns 30–60 bps long in combination with our results puts doubt on their existence. A further analysis of 30 selected introns in this range only verified two introns of 59 bp, and the true smallest introns in the *Arabidopsis* genome are probably close to this size. That there are many more introns 60–69 bp than 50–59 bp long (fig. 1) also supports this view. As to the mechanism of intron splicing, if an intron is too small (for example, smaller than 30 bp), it may hinder the splicing process. Or the sequence may be too short for the large spliceosome complex to process. In conclusion, our study may also provide insights into the working mechanism of spliceosome.

## Author Contributions

H.G. and W.C. designed the study; W.C., Y.Z., and X.M. carried out the experiments; W.C., Y.Z., X.M., and H.G. analyzed the data; W.C., C.A., and H.G. prepared the manuscript.

## Supplementary Material

Supplementary Data
