# Supplementary material for: The Putative Smallest Introns in the Arabidopsis Genome
Source: Genome Biol Evol. 2018 Sep 1;10(9):2551–7. doi: 10.1093/gbe/evy197 (PMC6161759; doi:10.1093/gbe/evy197)
Supplement: Supplementary Data [file evy197_supp.docx]

**Supplementary Materials:**

**Table S1**. Microarray data of the genes analyzed in this study

| **No.** | **Predicted intron** | **Length**  **(bp)** | **Sequencing results** | **Expression level** | | |
| --- | --- | --- | --- | --- | --- | --- |
|  |  |  |  | **Range** | **Average** | **Median** |
| 1 | AT1G62580.1-5 | 27 | cDNA | no data | no data | no data |
| 2 | AT2G04395.1-2 | 29 | cDNA | no data | no data | no data |
| 3 | AT5G51795.1-2 | 28 | gDNA | no data | no data | no data |
| 4 | AT2G07240.1-4 | 30 | gDNA | 3~10 | 3.852381 | 3.6907 |
| 5 | AT2G21330.3-6 | 30 | cDNA | 5~7921 | 2885.123 | 2243.161 |
| 6 | AT2G44980.1-10 | 30 | cDNA | 14~331 | 64.83128 | 44.9176 |
| 7 | AT5G50080.1-1 | 27 | cDNA | no data | no data | no data |
| 8 | AT3G53740.1-3 | 27 | cDNA | 1027~12159 | 6067.462 | 5935.961 |
| 9 | AT2G41700.2-18 | 30 | cDNA | 72~537 | 198.9268 | 167.0583 |
| 10 | AT2G31370.5-6 | 28 | cDNA | 21~313 | 184.8442 | 181.5199 |
| 11 | AT1G51490.1-10 | 23 | cDNA | 6~790 | 20.51011 | 7.7734 |
| 12 | AT3G51260.2-3 | 21 | cDNA | 175~1930 | 1096.329 | 1081.817 |
| 13 | AT3G55280.3-3 | 18 | cDNA | 322~5178 | 2156.039 | 1998.067 |
| 14 | AT4G35300.3-3 | 30 | cDNA | 11~588 | 230.9958 | 199.2358 |
| 15 | AT1G01620.2-1 | 29 | cDNA | 64~9345 | 4084.758 | 3695.914 |
| 16 | AT3G53980.2-2 | 25 | cDNA | 5~4413 | 341.6806 | 34.6293 |
| 17 | AT3G59350.3-6 | 23 | cDNA | 82~9318 | 762.6963 | 361.4451 |
| 18 | AT2G05520.2-2 | 21 | cDNA | 16~19862 | 5942.03 | 3999.611 |
| 19 | AT2G10930.1-1 | 29 | gDNA | 17~59 | 28.60344 | 27.6967 |
| 20 | AT4G38300.1-2 | 28 | cDNA | 7~99 | 19.64568 | 16.0636 |
| 21 | AT1G71280.1-2 | 25 | cDNA | 6~29 | 7.506363 | 6.7766 |
| 22 | AT3G28170.1-1 | 10 | gDNA | 14~47 | 19.35453 | 18.5473 |
| 23 | AT1G18050.1-3 | 8 | cDNA | 4~31 | 5.483333 | 4.9978 |
| 24 | AT5G22050.1-7 | 20 | gDNA | no data | no data | no data |
| 25 | AT2G40920.2-1 | 16 | cDNA | no data | no data | no data |
| 26 | AT1G27290.2-2 | 16 | cDNA | 95~2123 | 962.0365 | 922.2442 |
| 27 | AT1G02950.3-4 | 15 | cDNA | 12~309 | 61.53846 | 38.1686 |
| 28 | AT1G31170.3-5 | 15 | cDNA | 66~2454 | 938.5365 | 786.727 |
| 29 | AT5G48760.2-1 | 13 | cDNA | 149~2853 | 1167.083 | 942.5671 |
| 30 | AT2G14720.2-1 | 10 | cDNA | no data | no data | no data |
| 31 | AT5G30341.1-1 | 30 | cDNA | no data | no data | no data |
| 32 | AT4G06479.1-1 | 29 | gDNA | no data | no data | no data |
| 33 | AT2G13125.1-1 | 29 | gDNA | no data | no data | no data |
| 34 | AT2G06500.1-1 | 29 | gDNA | 9~40 | 15.56339 | 14.658 |
| 35 | AT1G49015.1-2 | 29 | gDNA | 4~6 | 4.661006 | 4.5465 |
| 36 | AT3G28020.1-4 | 28 | gDNA | 43~219 | 52.72608 | 50.3556 |
| 37 | AT1G76720.1-13 | 26 | gDNA | 22~290 | 74.50854 | 68.5433 |
| 38 | AT2G18530.1-2 | 24 | gDNA | 19~127 | 31.4942 | 31.7309 |
| 39 | AT2G24340.1-3 | 24 | gDNA | 6~33 | 9.469118 | 8.4244 |
| 40 | AT3G27600.1-1 | 23 | gDNA | no data | no data | no data |
| 41 | AT2G13125.1-2 | 23 | gDNA | no data | no data | no data |
| 42 | AT2G11010.1-4 | 23 | gDNA | 3~7 | 3.52722 | 3.33 |
| 43 | AT1G35860.1-1 | 23 | gDNA | 7~16 | 8.565616 | 8.2208 |
| 44 | AT2G05440.4-2 | 21 | cDNA | no data | no data | no data |
| 45 | AT3G05450.1-1 | 19 | cDNA | no data | no data | no data |
| 46 | AT1G72270.1-13 | 18 | cDNA | 10~28 | 13.6748 | 12.7476 |
| 47 | AT4G13850.2-5 | 15 | cDNA | 732~7855 | 3477.369 | 2829.572 |
| 48 | AT1G24460.1-4 | 14 | cDNA | 44~336 | 94.59856 | 77.8458 |
| S1 | AT1G76530.1~5 | 31 | cDNA | 4.65~100.27 | 9.867446 | 6.562 |
| S2 | AT4G01780.1~2 | 32 | gDNA | 49.31~1578.06 | 383.3458 | 231.3161 |
| S3 | AT4G28670.1~3 | 34 | cDNA | 5.28~475.76 | 13.02414 | 6.3644 |
| S4 | AT4G20900.1~4 | 35 | cDNA | 5.65~33.74 | 7.782778 | 6.9978 |
| S5 | AT5G07510.2~2 | 36 | cDNA | 22.64~3352.30 | 243.9753 | 33.9707 |
| S6 | AT2G36010.2~1 | 36 | cDNA | 9.85~230.06 | 76.33434 | 51.4305 |
| S7 | AT3G56300.1~5 | 37 | cDNA | 18.9~70.67 | 39.14814 | 34.3242 |
| S8 | AT1G02670.1~5 | 37 | cDNA | 16.18~138.11 | 41.20536 | 32.9777 |
| S9 | AT1G16150.1~2 | 38 | cDNA | 9.59~97.34 | 18.50296 | 13.8989 |
| S10 | AT1G14390.1~4 | 39 | cDNA | 5.97~47.95 | 9.020023 | 7.2198 |
| S11 | AT3G13920.2~5 | 41 | cDNA | 1354.4~6371.07 | 371.358 | 3606.78 |
| S12 | AT4G04710.1~3 | 43 | cDNA | 92.69~851.47 | 444.1073 | 455.1813 |
| S13 | AT5G40600.1~1 | 44 | cDNA | 15.13~116.52 | 50.20529 | 48.4681 |
| S14 | AT1G19090.1~3 | 44 | cDNA | 4.36~1485.93 | 24.86168 | 4.8132 |
| S15 | AT4G04680.1~4 | 45 | cDNA | no data | no data | no data |
| S16 | AT1G48740.1~4 | 45 | cDNA | no data | no data | no data |
| S17 | AT3G11040.1~9 | 45 | cDNA | 8.99~224.64 | 62.24768 | 51.665 |
| S18 | AT2G35075.1~2 | 46 | gDNA | 5.86~13.30 | 7.117949 | 6.8058 |
| S19 | AT4G15300.1~3 | 46 | cDNA | 6.03~21.86 | 8.541186 | 7.8581 |
| S20 | AT4G14310.2~2 | 47 | cDNA | 34.76~172.02 | 47.86 | 45.06 |
| S21 | AT3G43290.1~1 | 51 | gDNA | 24.97~74.77 | 35.31 | 33.87 |
| S22 | AT3G56160.1~1 | 52 | cDNA | 9.05~214.31 | 56.57791 | 55.9187 |
| S23 | AT3G09090.2~12 | 55 | cDNA | 88.46~527.51 | 285.221 | 277.4562 |
| S24 | AT1G15120.2~5 | 55 | cDNA | 620.02~5330.20 | 2356.172 | 2382.028 |
| S25 | AT4G12750.1~7 | 56 | cDNA | 38.21~272.46 | 126.2678266 | 113.6985 |
| S26 | AT4G21820.1~9 | 56 | cDNA | 7.73~238.38 | 55.53933 | 20.9804 |
| S27 | AT4G24930.1~3 | 59 | cDNA | 10.36~976.82 | 274.8812 | 184.703 |
| S28 | AT3G23080.2~4 | 59 | cDNA | 38.86~1450.84 | 497.7452 | 377.9675 |
| S29 | AT2G30650.1~3 | 59 | gDNA | 5.72~59.38 | 9.7593 | 7.286 |
| S30 | AT2G29390.1~5 | 59 | cDNA | 152.52~712.18 | 341.6107 | 333.2824 |

**Table S2**. Sequences of primer used in this study.

| **No.** | **Primers** | **Sequences** |
| --- | --- | --- |
| 1F | 1G62580-1 | 5'- CGA AAG GCG AAG TGA CCG -3' |
| 1R | 1G62580-3 | 5' - CGT CCC AAT CGT CCT TAT -3' |
| 2F | 2G04395-1 | 5'- CGA AGA AGA GAG ACA GTC CG -3' |
| 2R | 2G04395-4 | 5'- ATC CTC ACC TTC GAA CAA G -3' |
| 3F | 5G51795-3 | 5'- GCC ACT GTT GTC TAC AAT G -3' |
| 3R | 5G51795-2 | 5'- ATC TTC ACC ATC CCT CCT -3' |
| 4F | 2G07240-1 | 5'- GAA CCA ATC ATC TTC CCC -3' |
| 4R | 2G07240-2 | 5'- GAG ATG AAA CAG GAT TGG AG -3' |
| 5F | 2G21330-1 | 5'- CAA CGT CAT GTT CGA AGG -3' |
| 5R | 2G21330-2 | 5'-CTT CCT CAG ACT CGC CTT C-3' |
| 6F | 2G44980-1 | 5'- TGG TAA GCT TTT GGT TTT GG -3' |
| 6R | 2G44980-2 | 5'- CAG CAG CAA CAA GAT TCA AAC -3' |
| 7F | 5G50080-1 | 5'- CAT GGT CTC GGC CTT GAC -3' |
| 7R | 5G50080-2 | 5'- CCG CTG TAT CAA ACG TCC -3' |
| 8F | 3G53740-1 | 5'- ATG ACA ACT CCA CAA GTG AAG -3' |
| 8R | 3G53740-2 | 5'- CTC ATC TTG CGG AGA ACA C -3' |
| 9F | 2G41700-1 | 5'- CTT CTC CAA CTG TCT CTG TTG -3' |
| 9R | 2G41700-2 | 5'- CTT CCT GCT TAT CCT CGA TAT -3' |
| 10F | 2G31370-1 | 5'- TGG CTT GAC TGT TGA AAA C -3' |
| 10R | 2G31370-2 | 5'- CCA TTT TGT TGT TCT TGC TG -3' |
| 11F | 1G51490-1 | 5'- GGA TCA CTT GAT TTT CTA GGG -3' |
| 11R | 1G51490-2 | 5'- GAA TCT GAC GGA ATC CTG G -3' |
| 12F | 3G51260-1 | 5'- GGT ACA TTC TCT GCT TGG -3' |
| 12R | 3G51260-2 | 5'- CTT CGC AGG GCC TTT CTT G -3' |
| 13F | 5G40600-1 | 5'- CGA TTC CCG TTC CGT TAG -3' |
| 13R | 5G40600-2 | 5'- CTC ATC AAC TTC TGG AAT GG -3' |
| 14F | 4G35300-1 | 5'- CTC TTT CTA CCT TCA GAC ATG -3' |
| 14R | 4G35300-2 | 5'- GCT GAG GAA GAA CTA ATC CC -3' |
| 15F | 1G01620-1 | 5'- GCC ACT CTC ATT ACC CAT TG -3' |
| 15R | 1G01620-2 | 5'- CTG GAA GCC TTT GAC AAC -3' |
| 16F | 3G53980-1 | 5'- GTC TCT GCG CCA TTC TTC -3' |
| 16R | 3G53980-2 | 5'- ACA ACA ATC ACA CTG ATA CAC -3 |
| 17F | 3G59350-1 | 5'- GCT ACT CCA AGG CTA AGT G -3' |
| 17R | 3G59350-2 | 5'- GAA AGA GCA AAG AAG ACC CC -3' |
| 18F | 2G05520-1 | 5'- ATG CAT CAC TTG TTT CTC C -3' |
| 18R | 2G05520-2 | 5'- GTA ACT TCC CCC ACT TCC -3' |
| 19F | 2G10930-1 | 5'- ATG GAA GTG AGC GAT GG -3' |
| 19R | 2G10930-2 | 5'- GTG TTG TTC TGA CTC AAG TC -3' |
| 20F | 4G14310-1 | 5'- CTT AGA GCA AAC GAA GCC -3' |
| 20R | 4G14310-2 | 5'- CGT CCT CGA TGT GGA GTG -3' |
| 21F | 3G43290-1 | 5'- ATG ATC GAC GGT GAA GCA C -3' |
| 21R | 3G43290-2 | 5'- GCT TCC ATA CAC GGT AAT C -3' |
| 22F | 3G28170-1 | 5'- CTC TGA AGC GTT TTC ACA AGG -3' |
| 22R | 3G28170-2 | 5'- CTA GAA CAT CCG CTT GTA CC -3' |
| 23F | 1G18050-1 | 5'- GAA CTA GAG TTC GTT CGT TC -3' |
| 23R | 1G18050-2 | 5'- CAT ACT TGC GAG GTG GTG -3' |
| 24F | 5G22050-1 | 5'- CCG CGT CAA CAG ACA TCC -3' |
| 24R | 5G22050-2 | 5'- CAA GAC AAG TAC AAT GCT ACT -3' |
| 25F | 2G40920-1 | 5'- CCG TAT TCA AAA TCC AAA AGC -3' |
| 25R | 2G40920-2 | 5'- GAG ACG CAC TTG AAC CTC -3' |
| 26F | 1G27290-1 | 5'- GGT TTT CTA TGT CGC TGG C -3' |
| 26R | 1G27290-2 | 5'- CCA CAT GAT TTT CCC AAA CC -3' |
| 27F | 1G02950-1 | 5'- GGT TAG GTT TTG ATA CTC ACC -3' |
| 27R | 1G02950-2 | 5'- GAT TCG CAA GGC AAC AAA C -3' |
| 28F | 1G31170-1 | 5'- CTC GGG ATG TCA CAG ATA C -3' |
| 28R | 1G31170-2 | 5'- CTT TGA ACC ATT GTT TCA GC -3' |
| 29F | 5G48760-1 | 5'- CGA GAA ATA TCA TCA GCC G -3' |
| 29R | 5G48760-2 | 5'- GGA ACA AAC TTG AGA GCA TC -3' |
| 30F | 2G14720-1 | 5'- CAT CGA GCA CAT ACA AAG AG -3' |
| 30R | 2G14720-2 | 5'- GGC TCA TCA ACA TTA TCA GC -3' |
| 31F | 5G30341-1 | 5'- GGA CGG CGT GAT CAA GAG -3' |
| 31R | 5G30341-2 | 5'- CAC CAG TAG CAG AGA GGG -3' |
| 32F | 4G06479-1 | 5'- CAT GTT GAG TGG ATG ATC ACC -3' |
| 32R | 4G06479-2 | 5'- GTT CCT GTT CAA GTA GCA TC -3' |
| 33F | 2G13125-1 | 5'- GGG AGA GCC AGA AGA ACT TG -3' |
| 33R | 2G13125-2 | 5'- CTA GGA CCA AAC ACC TCC -3' |
| 34F | 2G06500-1 | 5'- GCA AAA GAG ACG AGA AGA G -3' |
| 34R | 2G06500-2 | 5'- GAC TCT AAC TCA GTC CAA CG -3' |
| 35F | 1G49015-1 | 5'- ATG GCT GGA GTA GAT GGT C -3' |
| 35R | 1G49015-2 | 5'- CCT TTT TAG GCT GGT TCT TG -3' |
| 36F | 3G28020-1 | 5'- CCA CTA CCA AGA TAA GGT CAG -3' |
| 36R | 3G28020-2 | 5'- CAC AAA TGC AGA AGG AAC AC -3' |
| 37F | 1G76720-1 | 5'- GCA TCA CAA AGA AAT AAA GGC -3' |
| 37R | 1G76720-2 | 5'- GCG CAG AAG ATT TTT ACT CC -3' |
| 38F | 2G18530-1 | 5'- CCG ATT TGG TAG TGT TGG TG -3' |
| 38R | 2G18530-2 | 5'- GCT TCA TAT TCT ACC TCC ACA -3' |
| 39F | 2G24340-1 | 5'- GGT TCT GGG CAC TAA GTC -3' |
| 39R | 2G24340-2 | 5'- CAC CTC CAA AAG TAC GAT C -3' |
| 40F | 3G27600-1 | 5'- GAT GAT GAT GAT GCT GAT GC -3' |
| 40R | 3G27600-2 | 5'- CAG TAA TTT CAG GTG GTG G -3' |
| 41F | 2G13125-1 | 5'- GGA GAG CCA GAA GAA CTT G -3' |
| 41R | 2G13125-2 | 5'- CTA GGA CCA AAC ACC TCC -3' |
| 42F | 2G11010-1 | 5'- GAG GCC AAT CAC AAA CTC -3' |
| 42R | 2G11010-2 | 5'- CGC GAT GAA TAG TAA GCA TG -3' |
| 43F | 1G35860-1 | 5'- GAT TGA GTA CTT GGG AAG TC -3' |
| 43R | 1G35860-2 | 5'- CTT GAC CTC AAC AAT TAC ACC -3' |
| 44F | 2G05440-1 | 5'- ATG GCT TCC AAG GCT TTG -3' |
| 44R | 2G05440-2 | 5'- CGT ATC CTC CTC CAC CAC -3' |
| 45F | 3G05450-1 | 5'- CTT CGA GCG ACG TAA ATG -3' |
| 45R | 3G05450-2 | 5'- CGT TCG CTT CAA GAT CGT AG -3' |
| 46F | 1G72270-1 | 5'- GTG AGA AAG TCC GTC AAA TTG -3' |
| 46R | 1G72270-2 | 5'- GCA TAT AAT CTC AAC TGG CG -3' |
| 47F | 4G13850-1 | 5'- GAG AGA CAG GAA GAT CAA GG -3' |
| 47R | 4G13850-2 | 5'- GAA ACC ACC ACC ACC ATC AC -3' |
| 48F | 1G24460-1 | 5'- CCG AGA TAT GGA GAA CAG G -3' |
| 48R | 1G24460-2 | 5'- CGT TGT TGT TCA TCA GAT GC -3' |
| S1F | 1G76530-1 | 5'- GTG GTT TAT GCC GGT TAA TG -3' |
| S1R | 1G76530-2 | 5'- CAG CTG TCA TCA TAG TTG G -3' |
| S2F | 4G01780-1 | 5'- GTG AGA GTG GAT CGA AGC -3' |
| S2R | 4G01780-2 | 5'- CAT TCT CTC GGT TCC ATT G -3' |
| S3F | 4G28670-1 | 5'- GCG AAC GCT AAA TGA TGA G -3' |
| S3R | 4G28670-2 | 5'- CTC ATT GAA GTT GTT GGT TGC -3' |
| S4F | 4G20900-1 | 5'- GTC AGG GAG AAT TGA AGA AG -3' |
| S4R | 4G20900-2 | 5'- GCT AAC ATT TCG ACG GCT C -3' |
| S5F | 5G07510-1 | 5'- GTG GTT ATT GCT GCC GTG -3' |
| S5R | 5G07510-2 | 5'- CTG GTC CGA ACA TTT TTG AG -3' |
| S6F | 2G36010-1 | 5'- CCA CCT ATA CGT CGT CAC -3' |
| S6R | 2G36010-2 | 5'- GAG GAG TTG ACT TGT TTC C -3' |
| S7F | 3G56300-1 | 5'- CAT TGA GAA TGG ATG TGG G -3' |
| S7R | 3G56300-2 | 5'- CCC ATC TTC ACA TTG TTG TTG -3' |
| S8F | 1G02670-1 | 5'- CAG GGT TCT TCA ATA CCA TG -3' |
| S8R | 1G02670-2 | 5'- CAG GAA TTG AGA GTG GTA AG -3' |
| S9F | 1G16150-1 | 5'- GTA TGG TTC CGA GAC GAT TTG -3' |
| S9R | 1G16150-2 | 5'- GAC ATC TCT ACA TTG CCT TC -3' |
| S10F | 1G14390-1 | 5'- GCA TTT GGT CAG TGT TCT TG -3' |
| S10R | 1G14390-2 | 5'- CAG CTG CTA TTA CTT TGC C -3' |
| S11F | 3G13920-1 | 5'- GAG AAG GTC ATG AGG GCC -3' |
| S11R | 3G13920-2 | 5'- CTA GTA CGG CAG AGC AAA C -3' |
| S12F | 4G04710-1 | 5'- GCT CCT GAA GTA TTA GAG GG -3' |
| S12R | 4G04710-2 | 5'- GTT CAT TGC TCG GAA TTG C -3' |
| S13F | 5G40600-1 | 5'- CGA TTC CCG TTC CGT TAG -3' |
| S13R | 5G40600-2 | 5'- CTC ATC AAC TTC TGG AAT GG -3' |
| S14F | 1G19090-1 | 5'- CAC ATT TGA GTG ATC GTG ATG -3' |
| S14R | 1G19090-2 | 5'- GAC CTT CAA TGC TGC AAC -3' |
| S15F | 4G04680-1 | 5'- GAT AAC CAG AGC CCT ATG G -3' |
| S15R | 4G04680-2 | 5'- GGT TAT GCG ATG GTG ACT G -3' |
| S16F | 1G48740-1 | 5'- CCA ATC AGG AGA CCG TAC C -3' |
| S16R | 1G48740-2 | 5'- GGC TTT ACG TCT GTT CGC -3' |
| S17F | 3G11040-1 | 5'- GGC TTT GGT TAC CAT GTT TC -3' |
| S17R | 3G11040-2 | 5'- GAG TAG GAG ATT GTG ATG GG -3' |
| S18F | 2G35075-1 | 5'- ATG GGT ATC AAT GAG TTT AGC -3' |
| S18R | 2G35075-2 | 5'- GCA ACT TAA CCT CGG CAC -3' |
| S19F | 4G15300-1 | 5'- CAA GGA GTC CCA TAA ACA TG -3' |
| S19R | 4G15300-2 | 5'- GTA CTC GAC CGC ATT CTC -3' |
| S20F | 4G14310-1 | 5'- CTT AGA GCA AAC GAA GCC -3' |
| S20R | 4G14310-2 | 5'- CGT CCT CGA TGT GGA GTG -3' |
| S21F | 3G43290-1 | 5'- ATG ATC GAC GGT GAA GCA C -3' |
| S21R | 3G43290-2 | 5'- GCT TCC ATA CAC GGT AAT C -3' |
| S22F | 3G56160-1 | 5'- CGA TAG CTA GTA CCC TGG -3' |
| S22R | 3G56160-2 | 5'- CCG CAT GTG CTG ATC TTA G -3' |
| S23F | 3G09090-1 | 5'- GGC TGA TAA TGT TGA CGG -3' |
| S23R | 3G09090-2 | 5'- GAT CTG GCT CTG CGT TAT C -3' |
| S24F | 1G15120-1 | 5'- GGC AGA TGA TGA AGT TGT TG -3' |
| S24R | 1G15120-2 | 5'- CCA CAT GTA GCA AAC TCA AG -3' |
| S25F | 4G12750-1 | 5'- GAA AAC TGT GAG CTT GCC -3' |
| S25R | 4G12750-2 | 5'- CCG CGT GAA CTC GTC TAG -3' |
| S26F | 4G21820-1 | 5'- GCA GTA GTT AGC AAC CAA AG -3' |
| S26R | 4G21820-2 | 5'- CTC GTT GGG AAT CCG TGG -3' |
| S27F | 4G24930-1 | 5'- CCT TGC CCT TCC ACG AAC -3' |
| S27R | 4G24930-2 | 5'- GGT AGG AGC ATA CCA ACC -3' |
| S28F | 3G23080-1 | 5'- GGT CCT GTT GTT TAT CGT AG -3' |
| S28R | 3G23080-2 | 5'- GGT ACG CCC TTA AGC CAG -3' |
| S29F | 2G30650-1 | 5'- GCA ACT GTT CCT TGC ATA TG -3' |
| S29R | 2G30650-2 | 5'- CTG AGG TTT TCT ATA CGT GG -3' |
| S30F | 2G29390-1 | 5'- GTA CGC GAC ACC GTT TGG -3' |
| S30R | 2G29390-2 | 5'- CAG GTT TCT TTT AGG GCC -3' |
